# Response to Powell and Ebel

**DOI:** 10.1017/S0950268826102064

**Published:** 2026-10-14

**Authors:** Fernando Sampedro, Craig W. Hedberg

**Affiliations:** School of Public Health, University of Minnesota, USA

We thank Powell and Ebel for pointing out the mistake in integrating the temperature values in the histogram. The correct minimum temperature should be 62.2°C when only using non-zero probabilities (cells E7:E20). It is not feasible to have a medium or medium-well burger at 53°C; the temperature profile was therefore constrained so that lower temperatures, which would correspond to rare burgers, were not included. This will be included in a corrigendum.

Sampedro et al. [1] report that their baseline model (with the error in at-home cooking temperature) estimated a mean annual number of Salmonella cases of 16036, with 87.8% of the predicted illnesses resulting from preparation and consumption at home (Table 2). Correcting the spreadsheet error in the at-home cooking temperature model input increases the estimated number of *Salmonella* illnesses due to consumption of ground turkey under the baseline scenario to a mean annual number of 204655. In comparison, the epidemiologic estimate of the annual *Salmonella* illnesses attributed to ground turkey is reported to be in the range of 17500 (p. 7). The discrepancy between the baseline prediction of the corrected spreadsheet and the epidemiologic estimate is greater than an order of magnitude, indicating major problems embedded in the model. The overestimation of illnesses is particularly problematic given that the model contains no microbial growth component, and all FSIS raw ground turkey samples below the limit of detection (not detected in 325 g) were deemed to be negative and not included in the analysis as left-censored data (p. 2).

As stated in the first comment, the correct minimum temperature should be 62.2°C when only using non-zero probabilities. Because the cells below 62.2°C (E1:E6) were assigned zero probability, they contribute no draws to the simulated distribution. The baseline estimate is therefore unchanged, and the figure of 204655 illnesses arises from assigning non-zero probability to cooking temperatures that were deliberately excluded from the model. We agree with Powell and Ebel that a potential limitation of the study is not accounting for *Salmonella* growth in ground turkey during temperature abuse. However, the impact on the number of illnesses should be minimal. A publication by Ingham et al. [2] showed lag phase durations of several *Salmonella* serovars in ground of 1364, 887, and 826 min (22.7, 14.8, and 13.8 h, respectively) at 10.0°C, 12.8°C, and 15.6°C (50°F, 55°F, and 60°F, respectively). These lag phase durations are longer than, or comparable to, the duration of the temperature abuse that could plausibly occur during transport home or during thawing of ground turkey. Additional text has been added to the corrigendum to state that *Salmonella* growth during potential temperature abuse was not included in the model. Mean serving size was simulated as 117 g with a maximum of 170 g. Serving sizes are below the 325 g FSIS sample size. This was the rationale for not including samples below the LOD, as the serving size would have <1 CFU/serving.

We thank Powell and Ebel for pointing out the mistake in calculating the percentage of diverted lots. We have recalculated the percentage of diverted lots and the number of illnesses after removing lots with different concentration thresholds. The revised calculations are very similar to those in the original publication, and thus no further changes are needed in the conclusion section. The revised calculations and revised Table 4 will be included in the corrigendum.

Each scenario (removing lots with a certain concentration) was modelled separately from the baseline by replacing the prevalence and concentration values reported earlier. The use of random or fixed seed values has advantages and disadvantages in a Monte Carlo simulation. Fixed seed values are better for replicating the results of your model since they are consistent every time you run the simulation. However, it might overlook variability that would occur naturally in real-world scenarios if you always use the same initial conditions. Random seeds, however, simulate the inherent randomness of real-world processes, capturing a broader range of possible outcomes and helping evaluate the stability of conclusions under varied conditions. We decided to use the latter approach due to the nature of the population-based risk assessment model. The findings reported in the letter on the randomness of using different initial seed values (7.8% and 9%) are much lower than the reduction effect observed under the different scenarios (38.5% for >10 MPN/g, 72.1% for >1 MPN/g, and 96.6% for ≥1 MPN/25 g) and thus do not affect the validity of the net effect of the different strategies evaluated. We included a convergence criterion (a change of <1% in the mean number of illnesses for both low- and high-virulence serotypes at the 95% confidence level). The convergence criterion has been added to the corrigendum.

We have responded to all the concerns raised and have clarified the model structure and the modelling approach. Changes applied in response to these comments will be included as a corrigendum.
